# Evaluation of the hyplex^® ^TBC PCR test for detection of *Mycobacterium tuberculosis *complex in clinical samples

**DOI:** 10.1186/1471-2180-10-95

**Published:** 2010-03-31

**Authors:** Sabine Hofmann-Thiel, Laziz Turaev, Harald Hoffmann

**Affiliations:** 1 IML red, Supranational Reference Laboratory of Tuberculosis, Asklepios Fachkliniken München-Gauting, Robert-Koch-Allee 2, 82131 Gauting, Germany; 2 National Reference Laboratory of Tuberculosis, Tashkent, Uzbekistan

## Abstract

**Background:**

Tuberculosis (TB) is one of the major public health concerns worldwide. The detection of the pathogen *Mycobacterium tuberculosis *complex (MTBC) as early as possible has a great impact on the effective control of the spread of the disease. In our study, we evaluated the hyplex^® ^TBC PCR test (BAG Health Care GmbH), a novel assay using a nucleic acid amplification technique (NAAT) with reverse hybridisation and ELISA read out for the rapid detection of *M. tuberculosis *directly in clinical samples.

**Results:**

A total of 581 respiratory and non-respiratory specimens from our pneumological hospital and the National TB Institute of Uzbekistan were used for the evaluation of the PCR assay. Of these, 292 were classified as TB samples and 289 as non-TB samples based on the results of the TB cultures as reference method. The PCR results were initially used to optimise the cut-off value of the hyplex^® ^TBC test system by means of a ROC analysis. The overall sensitivity of the assay was determined to be 83.1%. In smear-positive TB samples, the sensitivity of the hyplex^® ^TBC PCR test was estimated to 93.4% versus 45.1% in smear-negative samples. The specificity of the test was 99.25%. Of the two specimens (0.75%) with false-positive PCR results, one yielded a culture positive for non-tuberculous mycobacteria. Based on the assumption of a prevalence of 8% TB positives among the samples in our diagnostic TB laboratory, the positive and negative predictive values were estimated to 90.4% and 98.5%, respectively.

**Conclusions:**

The hyplex^® ^TBC PCR test is an accurate NAAT assay for a rapid and reliable detection of *M. tuberculosis *in various respiratory and non-respiratory specimens. Compared to many other conventional NAAT assays, the hyplex^® ^TBC PCR test is in a low price segment which makes it an attractive option for developing and emerging countries with high TB burdens.

## Background

Worldwide, tuberculosis (TB) remains one of the leading infectious diseases, accounting for nearly 3 millions deaths and over 8 million new cases annually [1]. The vast majority of TB cases occur in developing or emerging countries, particularly in Africa, South-East-Asia and the countries of the former Soviet-Union. Among them are up to 20% multidrug-resistant strains of *Mycobacterium tuberculosis *(MTB) [2]. In the control of the spread of TB, accurate and early laboratory diagnosis plays an important role.

Diagnosis of TB relies on the detection of acid-fast bacilli (AFB) by microscopy (smear) and culture followed by identification of isolates [3]. Microscopy is rapid and inexpensive but has a low sensitivity (10^4 ^to 10^5 ^AFB per ml). Culture is slow but more sensitive, detecting as few as 10^2 ^TB bacilli per ml. So far, culture is considered the "gold standard" for laboratory confirmation of TB. The main disadvantage is its slowness and therewith the delay in diagnostic of TB of up to several weeks.

A major breakthrough in diagnosis of TB was therefore achieved by the introduction of nucleic acid amplification techniques (NAAT) to detect *M. tuberculosis *complex (MTBC) directly in clinical specimens, which can give results within one day. Compared to microscopy, the value of NAAT lies (i) in its greater positive predictive values with smear-positive specimens in settings in which non-tuberculous mycobacteria are common, and (ii) in the possibility to rapidly confirm the presence of MTB in 50 - 80% of smear-negative TB cases [4,5]. Thus, compared to culture, NAAT can detect the presence of MTB weeks earlier for 80 - 90% of patients suspected to have pulmonary TB. These advantages can significantly improve patient care and TB control efforts.

There are currently several commercial NAAT methods available of which each uses a different method to amplify specific nucleic acid sequences of MTBC. These include, for example, the Roche COBAS Amplicor MTB test, the GenProbe Amplified *M. tuberculosis *Direct test (AMTD), the BD ProbeTec-ET or the Hain GenoType Mycobacteria Direct assay (GTMD). Available real-time polymerase chain reactions (PCR) systems are, for example, the Roche COBAS TaqMan MTB (CTM) test and the Cepheid Xpert MTB test. A series of evaluation studies [6-16] have analysed and compared the accuracy of commercial NAATs in both pulmonary and extrapulmonary TB. They show that most of the NAATs have high and consistent specificity and good positive predictive values but modest and variable sensitivity, particularly in smear-negative and extra-pulmonary TB.

An important issue is the implementation of NAATs in developing countries with high TB burden. However, prizes of commercial kits including required precision instruments are not affordable for most of the countries with high TB burden. Therefore, many of these countries use poorly validated in-house PCRs which show more variability in their accuracy [17]. There is a high demand for well validated, affordable commercial NAATs for use in low-resource countries.

A novel commercial NAAT, which meets the demands for a low cost system, has been recently introduced. The hyplex^® ^TBC test (BAG Health Care, Lich, Germany) is a qualitative system for the detection of members of the MTBC and is based on multiplex PCR followed by reverse hybridisation to specific oligonucleotide probes and ELISA detection.

In the present study we performed a clinical evaluation of the hyplex^® ^TBC test using well-characterised TB and non-TB samples. PCR data were compared to the results of conventional microscopy and culture techniques. Finally, the potential impact of hyplex^® ^TBC test on laboratory diagnostics of TB was assessed.

## Results

In the present study, we performed a comprehensive clinical evaluation of the hyplex^® ^TBC PCR in order to estimate and optimise its diagnostic potential. A total of 581 clinical specimens from our frozen archive were included comprising 292 TB samples and 289 non-TB samples (Table 1).

**Table 1:** Classification of samples

Clinical group	Samples (n)
*TB POSITIVE*	*292*

1. infection with *M. tuberculosis*, culture and smear positive	230

2. infection with *M. tuberculosis*, culture positive, smear negative	62

*TB NEGATIVE*	*289*

3. no TB	269

4. no TB but culture positive for non-tuberculous mycobacteria	20

*TOTAL*	*581*

### Cut-off validation

**Figure 1:**
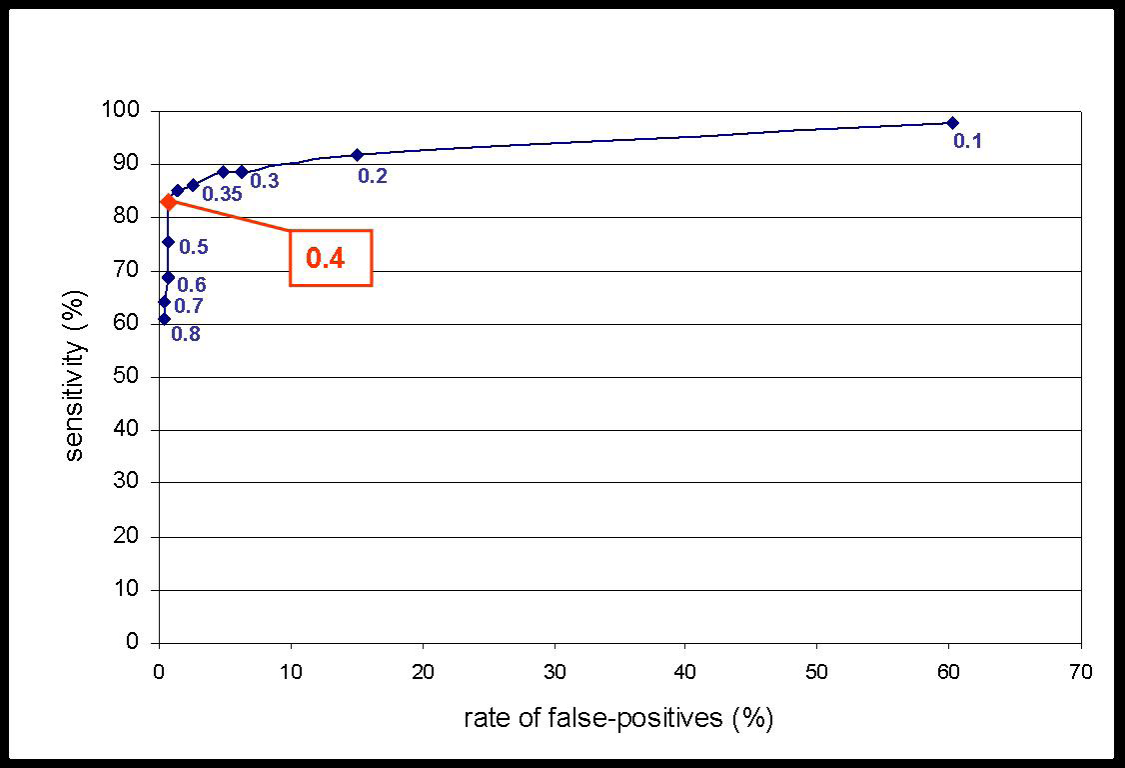
**ROC curve analysis**. Based on the clinical classification of specimens into TB or non-TB, hyplex^® ^TBC results were analysed at different cut-off values regarding the diagnostic performance. Therefore, the rate of false-positive PCR results (100% minus specificity) was plotted against the sensitivity at cut-off values of 0.100, 0.200, 0.300,0.325, 0.350, 0.375, 0.400, 0.500, 0.700 and 0.800, corresponding to the optical densities of the ELISA read-out.

The read-out end-point of the hyplex^® ^TBC test is an optical density (OD) value of the ELISA after reverse hybridisation. In an initial step, we determined the best cut-off value for the discrimination of TB and non-TB specimens by means of a ROC (receiver operating characteristic) curve analysis. Therefore, the sensitivity of the test was determined for each potential cut-off value between 0.100 and 0.800 and plotted against the rate of false positive results (Figure 1). The criteria of the best cut-off were defined as (i) a false-positive rate as low as possible ranging at least below 1% in order to minimise the risk of the false diagnosis of a TB, and (ii) a sensitivity as high as possible. The optimal cut-value was set to an OD of 0.400, where the false-positive rate was 0.75% with sensitivity over 80% considering all specimens.

### Inhibition rate

The version of the hyplex^® ^TBC test used in this study contained hybridisation modules for an internal control (IC) allowing for the detection of inhibitors of the PCR amplification. In general, samples with an OD_IC _< 0.300 were considered as inhibited as long as the TBC PCR was negative (OD_TBC _< 0.400). Twenty-four out of the 581 samples (4.1%) were excluded from further analysis due to inhibition of the test reaction (Table 2). A higher rate of inhibition was found in the non-TB group (7.6%) compared to the TB group (0.7%). When looking at the different types of specimens, the highest rate of inhibition was found with urine samples (16.3%). Among samples of respiratory origin, bronchial/tracheal secretes showed the highest rate of inhibition (5.9%), followed by bronchoalveolar lavage (BAL) (4.0%) and sputum (2.4%) (Table 2).

**Table 2:** Rate of inhibition

	specimens (n)	inhibited specimens (n)	rate of inhibition (%)
*ORIGIN OF SAMPLE*			

Sputum	374	9	2.4

Bronchial secrete	85	5	5.9

BAL	50	2	4.0

Urine	43	7	16.3

Punctuates/fluids	28	1	3.6

Biopsies	1	0	0

*CLINICAL GROUP*			

TB	292	2	0.7

non-TB	289	22	7.6

*TOTAL*	*581*	*24*	*4.1*

### Sensitivity

Of the remaining 557 samples without inhibitors, 290 were classified as TB samples based on the detection of MTB in culture (Table 3). Of these, 228 (79%) were smear-positive and 62 (21%) were smear-negative. 267 of 557 samples were considered as non-TB group based on negative cultures for MTB. Among these, culture of 20 samples revealed non-tuberculous mycobacteria (5 × *M. intracellulare*, 5 × *M. gordonae*, 4 × *M. avium*, 3 × *M. celatum*, and 1 × each *M. mucogenicum*, *M. interjectum *and *M. kansasii*). Overall, 243 of 557 (43.6%) yielded positive PCR results (OD ≥ 0.400; median OD value: 1.32), and 314 negative results (OD < 0.400; median OD value: 0.147).

**Table 3:** Sensitivity and specificity of the hyplex^® ^TBC test

	PCR results				
	
	positive (n)	negative (n)	total (n)	sensitivity (%)	specificity (%)
*ALL SAMPLES*	*243*	*314*	*557*		

**TB samples**	**241**	**49**	**290**	**83.1**	

smear-positive	213	15	228	93.4	

smear-negative	28	34	62	45.1	

**non-TB samples**	**2**	**265**	**267**		**99.25**

non-NTM	1	246	247		99.5

NTM	1	19	20		95.0

*RESPIRATORY SAMPLES*	*237*	*257*	*494*		

**TB samples**	**234**	**44**	**278**	**84.2**	

smear-positive	210	14	224	93.7	

smear-negative	24	30	54	44.4	

**non-TB samples**	**2**	**213**	**215**		**99.1**

non-NTM	1	195	196		99.5

NTM	1	18	19		94.7

*NON RESPIRATORY SAMPLES*	*11*	*53*	*64*		

**TB samples**	**11**	**1**	**12**	**91.6**	

smear-positive	4	0	4	100	

smear-negative	7	1	8	87.5	

**non-TB samples**	**0**	**52**	**52**		**100**

non-NTM	0	51	51		100

NTM	0	1	1		100

Of the 290 TB culture positive samples, 241 gave positive PCR results yielding an overall sensitivity of 83.1% (Table 3). The sensitivity for smear-positive specimens (n = 228) was 93.4%, for smear-negative specimens (n = 62) 45.1%. Similar sensitivities were calculated considering respiratory TB specimens only (n = 278): the overall sensitivity was 84.2%; the sensitivities for smear-positive and smear-negative samples were 93.7% and 44.4%, respectively. Among non-respiratory samples, all smear-positive TB samples (n = 4) and seven out of eight smear-negative TB samples were detected by PCR (sensitivities of 100% and 87.5%, respectively).

### False negatives

Fifteen out of 228 culture and smear-positive TB samples (6.6%) were negative by hyplex^® ^TBC PCR (Table 3). Repeat testing of these false-negative samples also yielded negative results with hyplex^® ^TBC. The existence of inhibitors could be excluded in the samples by high OD_IC _values ranging from 1.5 to 2.2. Only one sample showed a somewhat lower OD_IC _which was however still above the cut-off (OD_IC _= 0.37) indicating the presence of some inhibiting factors which could have influenced the TB-specific PCR. All false-negative samples (n = 15) were re-assessed by the CTM PCR test, a real-time PCR system based on MTBC specific sequences within the 16S rRNA genes. Positive PCR results were obtained with all specimens tested but one (*data not shown*). These data indicate that a small proportion of TB positive samples (smear and culture positive) were indeed not recognized by the hyplex^® ^TBC system.

### Specificity

Of the 267 non-TB samples, 265 gave negative PCR results yielding a specificity of 99.25%. A specificity of 99.5% was obtained for non-TB samples excluding cases of infection with NTMs (n = 247). Considering NTM samples only (n = 20), the specificity was 95%. Similar values were obtained for respiratory non-TB samples (n = 215) (Table 3). Among the non-respiratory samples, all culture negative samples (n = 52) gave also negative PCR results (100% specificity).

### False positives

Seven out of the 267 MTB culture-negative specimens were initially hyplex^® ^TBC PCR positive and considered as false-positives. Assessment of these samples by CTM PCR gave negative results with all seven samples. Five of these samples were also clearly negative on repeat with the hyplex^® ^TBC PCR, while in two samples, the positive values of the first runs were confirmed on repeat. One of these two specimens gave a positive culture for *M. intracellulare*, the other one showed no mycobacterial growth on culture. Together, based on merged PCR data and culture results, two out of 267 MTB culture negative specimens (0.75%) were finally classified as false-positive hyplex^® ^TBC PCR results (Table 3).

### Positive and negative predictive values

Positive (PPV) and negative (NPV) predictive values largely depend on the prevalence of a disease. In particular, with low prevalence, the specificity of a test has to be very high, otherwise the PPV of the test will be poor. The proportion of TB samples (52%) included in this study was rather high and did not reflect the real situation of a TB diagnostic laboratory. In our laboratory, real time PCR (CTM PCR) yields between 7.0% and 9.5% positive results, depending on year and season. Assuming a mean rate of 8% of TB positive samples and a number of approximately 3000 PCR assays per year, the PPV of the hyplex^® ^TBC test would be calculated to 90.4%, and the NPV to 98.5% (Table 4), based on the sensitivity and specificity values found in this study (83.1% and 99.25%).

**Table 4:** Predictive values at cut-off values 0.400 and 0.200

	cut-off 0.400			cut-off 0.200		
	
	PCR pos^b^	PCR neg^b^	*TOTAL*^a^	PCR pos^b^	PCR neg^b^	*TOTAL*^a^
TB pos (n)	199	41	*240*	221	19	*240*

TB neg (n)	21	2739	*2760*	414	2346	*2760*

*TOTAL (n)*	*220*	*2780*	*3000*	*635*	*2365*	*3000*

PPV^c ^(%)	90.4			34.8		

NPV^c ^(%)	98.5			99.1		

^a ^Based on the assumption of a mean rate of 8.0% true TB positive specimens and a total number of 3000 specimens in a routine TB laboratory per year, the resulting numbers of TB positive and negative samples were calculated.

^b ^Based on the specificity and sensitivity values found in this study, the numbers of expected PCR positive and negative results among 3000 were calculated. Resulting numerical values were rounded.

^c ^Positive and negative predictive values were deduced from calculated PCR positive and negative results.

Finally, the validity of the hyplex^® ^TBC test was determined using an OD cut-off value for positive results of 0.200 as given in the instructions of the manufacturer. Using this value, the sensitivity of the test would rise to 92% while the specificity would decrease to 85% (*data not shown*). The PPV and NPV deduced from these sensitivity and specificity estimates would be calculated to 34.8% and 99.1%, respectively (Table 4). Thus, the PPV of the hyplex^® ^TBC test is dramatically decreasing when the cut-off is changed to OD 0.200, meaning that out of 1000 PCR-positive results only 348 truly indicate TB. Furthermore, the rate of false positives of 15% at cut-off of OD 0.200 would be above the acceptable limit.

## Discussion

The hyplex^® ^TBC PCR test is a new qualitative diagnostic NAAT system for the detection of MTBC in human specimens. Compared to most of the available commercial NAAT tests, which range from about 20 to 35 Euro (US$ 25 to 50) per test, it represents a low-cost system. Costs of the hyplex^® ^TBC test are estimated to ten to twelve Euro per test in industrialised countries. For developing countries, where most of the TB occurs, significantly lower prices can be considered. In contrast to real-time assays which require precision instruments as well as capacity to maintain these instruments, the hyplex^® ^TBC test can be applied in all laboratories with standard equipment for molecular biology techniques and, therefore, allows for the application also in low-budget laboratories, particularly in developing and emerging countries. However, the low costs of equipment and reagents go along with a significant increase in the hands-on time. Whereas highly automated tests like real-time assays may generate results within less than two hours with very low hands-on time, the hyplex^® ^TBC test requires multiple workstations for specimen preparation, target amplification and amplicon detection. Including column-based DNA preparation, the assay will take up to 6 hours to perform. This is comparable to other NAAT assays which are largely performed manually like, for example, the GTMD assay [16]. Similar to other NAAT assays, the hyplex^® ^TBC test is certainly suitable for partial automatation, for example by use of full automated systems for hybridisation and ELISA reading, which can significantly decrease the hands-on time of the test.

Initially, the hyplex^® ^TBC PCR test was validated by the manufacturer using a set of 40 clinical specimens (*data not shown*). In order to retrieve the highest sensitivity possible, the cut-off value was set to 0.200 in the manufacturer's instructions. This cut-off was technically controlled using DNA of different *Mycobacterium *and non-mycobacterial species. None of 96 different strains of different species other than *Mycobacterium *was positive (instruction for use, BAG Health Care). Out of 33 *Mycobacterium *strains, five MTBC strains (2 × MTB, 1 × *M. africanum*, 1 × *M. cannettii*, 1 × *M. bovis*) were positive. Twenty-eight NTM strains of 25 different species were tested and three (2 × *M. kansasii*, 1 × *M. gadium*) gave ELISA signals of about OD 0.300 that were considered positive following the instructions of the manufacturer. Thus, the "technical" sensitivity can theoretically be assumed 100%, while the technical specificity would be only 97.6% given a cut-off value of OD 0.200. Using the same cut-off, the sensitivity in our study set would be 92%, but the specificity would be as low as 85%, meaning that every seventh positive PCR result would be a false-positive one. However, the improved sensitivity by use of cut-off value 0.200 does not justify the risk of a false-positive diagnosis of TB in 15% of cases. Therefore, following our ROC analysis the optimal cut-off value of the hyplex^® ^TBC PCR assay was set to an OD of 0.400 in our study. Using this corrected value, the technical specificity determined by the manufacturer would indeed rise to 100%, while diagnostic sensitivity and specificity still range within reasonable limits.

The hyplex^® ^TBC offers an overall sensitivity of 83.1% and a specificity of 99.25%, when compared to culture results as standard reference. The overall sensitivity of 83.1% was similar to that found for other NAAT assays which tested respiratory and non-respiratory specimens (range: 61.8% to 93.5%; median: 83.5%) [7-10,12-16,18,19]. In contrast to some other studies which found significantly reduced sensitivities for non-respiratory specimens with various NAATs [7,10,14], the hyplex^® ^TBC assay even showed a higher sensitivity for non-respiratory samples (91.6% for non-respiratory *versus *84.2% for respiratory samples). Resolving against smear-negative specimens, the sensitivity of the hyplex^® ^TBC test was rather in the lower range (45.1%) when compared to other NAAT assays (range: 46% to 75,3%, median: 56%) [8,9,11-13,15,18-20]. Resolving against smear-positive specimens only, the sensitivity of the hyplex^® ^TBC test (93,4%) was in accordance with other NAAT assays (range: 91,7% to 100%; median: 96,2%) [8,11,13-15,18,19]. The overall specificity estimate of 99.25% for hyplex^® ^TBC was remarkably high compared to other NAAT assays (range: 97.4% to 100%; median: 99.2%) [7-9,11,14-16,18,20] and even ranged clearly above the pooled specificity of 97% found by meta-analysis [6].

The positive and negative predictive values (90.4% and 98.5%) were calculated from specificity and sensitivity estimates found in this study after extrapolation to a total number of 3000 specimens per year and a prevalence of true TB positive specimens of 8%. When compared to other evaluation studies which were based on similar rates of true TB positive samples (range: 10% to 13.2%) [8,11,21], the PPV of 90.4% of the hyplex^® ^TBC was in the lower third (range: 88.5% to 100%) whereas the NPV of 98.5% turned out excellent (range: 96.7% to 98.6%). In many studies, the prevalence of positive specimens in the respective setting of routine diagnostics was not included in the calculation of the PPV and NPV. This resulted mostly in an overestimation of the significance of the values. Additionally, the values are influenced by factors like the selection of specimens. For these reasons, the comparison of PPV and NPV with former studies and other assays is rather difficult.

Only two non-TB samples were finally classified as false-positive. In one of them grew *M. intracellulare*. It is unlikely that the positive PCR resulted from a dual infection of the patient with *M. intracellulare *and MTB. Furthermore, the absence of MTB DNA in this specimen was assessed by CTM PCR. Speculatively, some unspecific binding of the NTM amplification product to TBC-specific probes could have caused this false-positive result. False-positive PCR results due to sporadic cross-reactivity with non-tuberculous mycobacteria has been suspected earlier also with other NAAT systems [8,22,23]. As the technical validation of the hyplex^® ^TBC kit had indeed shown some unspecific binding for single *Mycobacterium *species, it would be possible also for the *M. intracellulare*. The second false-positive specimen originated from a case without a known MTB infection. It cannot be ruled out completely that very low amounts of MTB nucleic acids originating from an early TB infection may have led to positive PCR results with hyplex^® ^TBC.

Among smear-negative, culture-positive specimens, 34 out of 62 were not detected by hyplex^® ^TBC. This was, at least in part, due to the fact that the cut-off has been increased from OD 0.200 to OD 0.400 in order to reduce the false-positive rate to a minimum. It would certainly be worth trying, whether the sensitivity could be increased by applying higher volumes of sample. Our evaluation was performed with a sample volume of 10 μl, but theoretically sample volumes up to 40 μl can be applied. However, too much DNA may considerably reduce the effectiveness of a PCR and, in return, would lead to a higher rate of inhibition. The optimal volume of specimen needs to be determined in further investigations.

Seven percents of smear-positive, culture-positive samples also escaped the detection by hyplex^® ^TBC. It is unlikely that this was caused solely by too low amounts of MTB DNA, since most of these specimens yielded clearly positive smear microscopy results (at least between 10 and 50 acid fast bacilli per 100 fields) and re-assessment by CTM PCR gave positive results with 14 of 15 specimens. The hyplex^® ^TBC PCR is based on target sequences of a house keeping gene. It can be speculated that missing of some of these TB samples by hyplex^® ^TBC was related to single nucleotide polymorphisms within this gene. This question should be studied and the results may certainly help to optimise the oligonucleotide probes used in the kit.

## Conclusions

Hyplex^® ^TBC is an accurate and reliable NAAT assay for the direct detection of MTB in respiratory and non-respiratory specimens. Similar to other commercial NAATs, the hyplex^® ^TBC assay is impacted by the compromise between specificity and sensitivity: specificity is maximised at the cost of sensitivity. Compared to other commercial NAAT systems, the hyplex^® ^TBC assay shows excellent specificity estimates but slightly lower sensitivity, in particular for smear-negative TB specimens. Also, when the assay is used as rapid confirmation test for smear-positive specimens one should be aware of the fact that a small percentage of TB infections may be not detected. As it holds true also for other NAAT assays, hyplex^® ^TBC test cannot replace culture but should be always carefully interpreted along with clinical data and conventional tests.

## Methods

### Clinical specimens

A total of 581 clinical specimens, sent to our TB laboratory from April 2007 to October 2007, were taken from our frozen archive. 514 specimens were sent to our laboratory by German health centres for routine TB diagnostics. Further 67 samples were sent by the National DOTS centre of Uzbekistan to us as the supranational reference laboratory (SRLN) partner in the frame of the national TB survey.

292 specimens were classified as TB samples based on cultures being positive for MTB comprising 230 smear positive and 62 smear negative specimens. 289 specimens were classified as non-TB samples based on negative culture results. Among these, 20 samples were positive for NTMs (Table 1). The whole study set included 509 respiratory samples, 43 urine samples, 28 punctates and other fluid samples (pleural punctates, abscess fluids, gastric secretions, etc) as well as one tissue biopsy.

### Processing of samples

All specimens were decontaminated according to DIN 58943-3:2008. In brief, specimens were 1:1 mixed with *N*-acetyl-L-cysteine (NALC)-NaOH (final concentrations 1% NaOH, 0.7% NaCitrate, 0.25% *N*-acetyl-cysteine) and rotated for 20 min. After neutralisation with 0.5 M phosphate buffer (pH 6.8), and centrifugation (3000 × *g *for 20 min) in order to concentrate the mycobacteria, the sediment was resuspended in 1 ml phosphate buffer. Smears were prepared from this suspension and stained with auramin O following DIN 58943-32:2008. Fluorescence microscopy was performed with 400 × magnification. Of the sediment, 100 μl each were transferred to solid media (Loewenstein-Jensen, Stonebrink); 500 μl were inoculated into Mycobacteria Growth Indicator Tubes (MGIT™) (Becton-Dickenson, Heidelberg, Germany) and incubated in the Bactec™ MGIT 960 incubator according to the manual of the manufacturer. If demanded by the clinician, diagnostic PCR was performed using the CTM PCR test (Roche Diagnostics GmbH, Mannheim, Germany) following the instructions of the manufacturer. The leftover suspension (400 to 700 μl) was frozen at -60°C until further processing in the frame of the present study.

Media were incubated up to 8 weeks. In case a primary culture turned positive, the isolate was identified by DNA line probe assays (Genotype CM, Genotype MTBC, Hain Lifesciences, Nehren, Germany).

### Isolation of genomic DNA

DNA extraction was performed using the hyplex^® ^Prep module (BAG Health Care, Lich, Germany). In brief, 100 μl decontaminated, concentrated clinical sample was added to 200 μl lysis buffer and incubated at 99°C for 15 min. Following centrifugation, 200 μl of the supernatant was transferred to a new tube, mixed with binding buffer and loaded onto a hyplex^® ^Prep column. Further steps including washing of columns and elution in 100 μl elution buffer was done as recommended by the manufacturer.

### hyplex^® ^TBC PCR

The hyplex^® ^TBC test comprises the following work steps: (i) multiplex PCR of internal control (IC) sequences and genomic target sequences; (ii) heat-denaturation of amplification products; (iii) hybridisation to specific probes immobilised onto microtiter strips; and (iv) detection by ELISA followed by photometric measurement at 450 nm. Different from the commercially available version, the study version contained an internal control for the detection of inhibitors of the amplification of PCR products.

#### Amplification reaction

A 50 μl reaction volume contained 10 μl of sample lysate (or 10 μl negative/positive control included in the kit), 1 μl nucleotide mix, 2 μl primer mix, 5 μl 10 × PCR buffer, 0,4 μl Tth-DNA polymerase (5 U/μl) (BAG Health Care, Lich, Germany), and 31,6 μl PCR-grade water. Thermal cycling was as follows: 5 min at 94°C, then 45 cycles of 25 sec at 94°C, 25 sec at 52°C, 20 sec plus 1 sec/cycle at 72°C, and final extension of 3 min at 72°C. After completing of the PCR, reaction mixtures were used immediately for reverse hybridisation or stored at 4°C until use within the next 16 hours latest.

#### Reverse hybridisation and detection

After heat-denaturation (10 min at 95°C) of the PCR reaction mixture, 10 μl was immediately added to 100 μl pre-cooled hybridisation solution in new tubes and mixed thoroughly. 50 μl each was then quickly transferred by pipette to hybridisation cavities of the hyplex^® ^TBC and the hyplex^® ^IC module. After incubation of the microtiter plate for 30 min at 50°C, cavities were washed three times with 200 μl pre-warmed (50°C) stringent wash buffer followed by one washing step with normal wash buffer. Freshly prepared conjugate solution (100 μl) was added for 30 min at room temperature followed by three washing steps at room temperature with each 200 μl of washing buffer. 100 μl of substrate solution was then added to each well and after 15 min at room temperature the reaction was stopped with 100 μl stop solution. Measurement of the extinction of the individual wells was done in a microtiter photometer at 450 nm with a reference wave length of 620 - 650 nm.

### CTM PCR

Real-time PCR was performed on a COBAS^® ^TaqMan^®^48 according to the manufacturer's instructions using the COBAS^® ^TaqMan^® ^MTB kit (Roche Diagnostics, Mannheim, Germany) and 50 μl of DNA lysate. For routine laboratory diagnostics, lysis of decontaminated, concentrated specimens was performed using the AMPLICOR^® ^Respiratory Specimen Preparation Kit (Roche Diagnostics, Mannheim, Germany) comprising washing, lysis and neutralisation buffer. When using DNA isolated by the hyplex^® ^Prep Module as template, the DNA had to be mixed with appropriate volumes of lysis and neutralisation buffer prior to CTM PCR.

### Validation and analysis of data

Diagnostic culture was considered as the "gold standard". In those cases in which culture results were discrepant from the PCR results, hyplex^® ^TBC PCR was repeated and samples were re-tested with the Roche CTM test. Statistical data analyses were done using Epi Info™ Version 3.5.1 accessible by CDC (Center for Disease Control and Prevention).

## Competing interests

The authors declare that they have no competing interests.

## Authors' contributions

S.H.-T. participated in the design of the study, supervised the laboratory work, participated in data analysis and drafted the manuscript, L.T. conducted most of the experimental work, H.H. supervised the study, participated in its design, the data analysis and the preparation of the manuscripts. All authors read and approved the final manuscript.
